# Digital image quantification of siderophores on agar plates

**DOI:** 10.1016/j.dib.2016.01.054

**Published:** 2016-02-03

**Authors:** Megan Y. Andrews, Cara M. Santelli, Owen W. Duckworth

**Affiliations:** a Department of Soil Science, North Carolina State University, Raleigh, NC 27695-7619, USA; b University of Minnesota, Department of Earth Sciences, Minneapolis, MN 55455-0231, USA

## Abstract

This article presents visual image data and detailed methodology for the use of a new method for quantifying the exudation of siderophores during fungal growth. The data include images showing time series for calibration, fungal exudation, and negative controls, as well as replication accuracy information. In addition, we provide detailed protocols for making CAS assay layer plates, the digital analysis protocol for determining area of color change, and discuss growth media that do and do not work with the layer plate method. The results of these data, their interpretation, and further discussion can be found in Andrews et al., 2016 [1].

## Specifications table

**Table:** 

Subject area	*Biology*
More specific subject area	*Environmental biogeochemistry*
Type of data	*Images organized by figure.*
How data was acquired	*Photographs of agar plates taken using a camera and lightbox and subsequently digitally analyzed using JMicroVision 1.2.7*
Data format	*Analyzed digital images*
Experimental factors	*Photographs were background corrected and resized in Photoshop Elements prior to digital analysis.*
Experimental features	*CAS Layer plates were made with reduced toxicity to promote fungal growth and provide a method to observe and quantify siderophore exudation by fungal isolates.*
Data source location	*Raleigh, NC, USA*
Data accessibility	*Data is with this article.*

## **Value of the data**

• The data presented in this note details the method used to quantify siderophore equivalents exuded by fungi on agar-solidified growth plates [1].
• The visual data included illustrate the use of the CAS layer plate method to compare siderophore exudation patterns between different fungi, or between the same fungi on different media.
• The data presented here provide a new method to assess siderophore production in fungi, which can then be compared to other fungal experiments and aid in the design of future experiments.
• Details of media that were successful, and not successful, as part of these assay plates is presented as a starting point for testing other media with this assay method.

## 1. Data

**Fig. 1:**
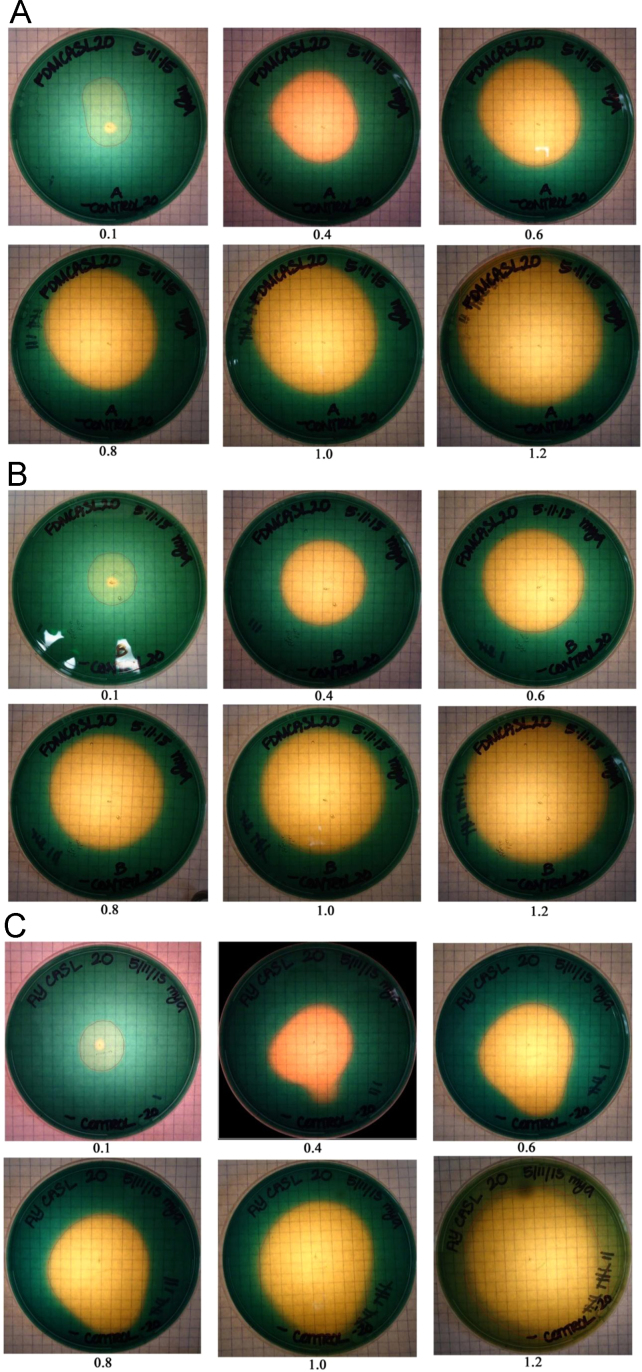
Images of CAS layer plates used to calibrate and verify siderophore quantification by serially pipetting micromoles of DFOB onto the center of the plate and allowing it to diffuse over 24 h. Beneath each photo, the number indicates the amount (µmol) of DFOB added. The CAS layer plates shown used two different agar-solidified growth media: (A and B) Fungal Defined Media (FDM) and (C) Acetate–Yeast (AY) media. The red lines indicate the yellow portion of the plate selected during the digital analysis protocol.

**Fig. 2:**
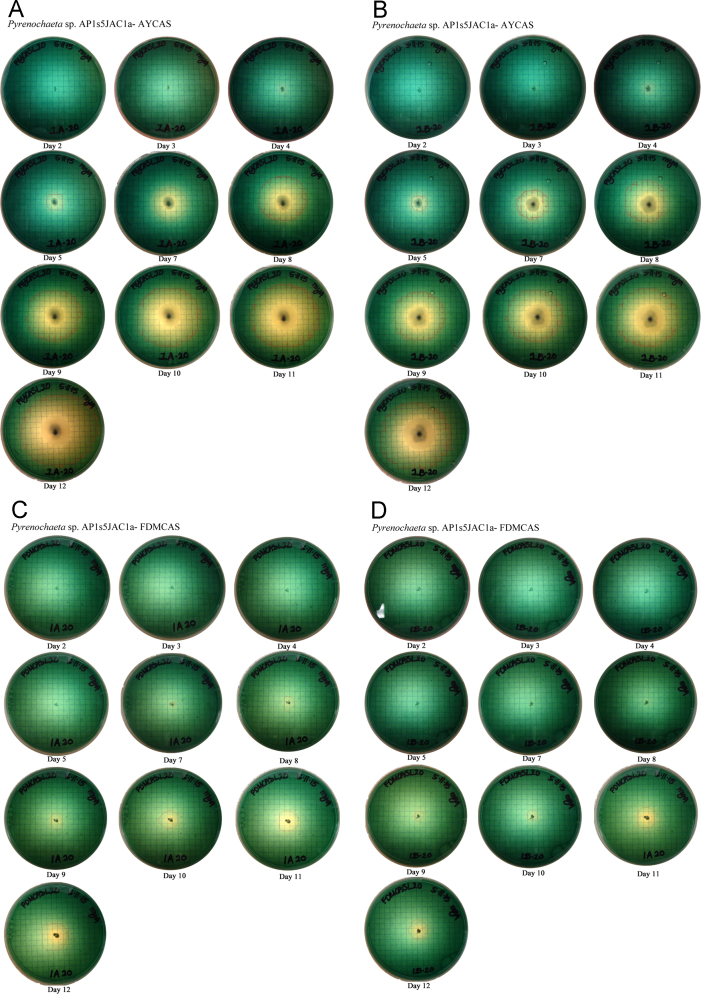
Image files for *Pyrenochaeta* sp. AP1s5JAC1a grown on (A and B) Acetate–Yeast (AY) based CAS layer plates and (C and D) Fungal Defined Media (FDM) media based CAS layer plates. The text below each image indicates the time elapsed in days since inoculation. A. *Pyrenochaeta* sp. AP1s5JAC1a – AYCAS. B. *Pyrenochaeta* sp. AP1s5JAC1a – AYCAS. C. *Pyrenochaeta* sp. AP1s5JAC1a – FDMCAS. D. *Pyrenochaeta* sp. AP1s5JAC1a – FDMCAS.

**Fig. 3:**
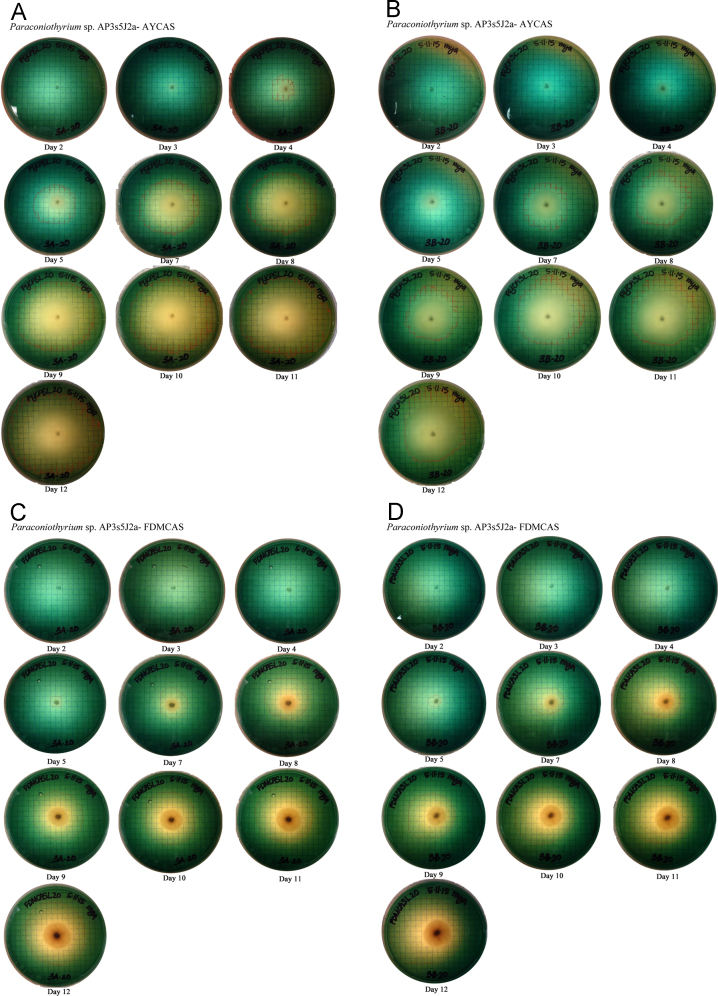
Image files of *Paraconiothyrium* sp. AP3s5J2a grown on (A and B) Acetate–Yeast (AY) based CAS layer plates and (C &D) Fungal Defined Media (FDM) media based CAS layer plates. The text below each image indicates the time in days since inoculation. A. *Paraconiothyrium* sp. AP3s5J2a – AYCAS. B. *Paraconiothyrium* sp. AP3s5J2a – AYCAS. C. *Paraconiothyrium* sp. AP3s5J2a – FDMCAS. D. *Paraconiothyrium* sp. AP3s5J2a – FDMCAS.

**Fig. 4:**
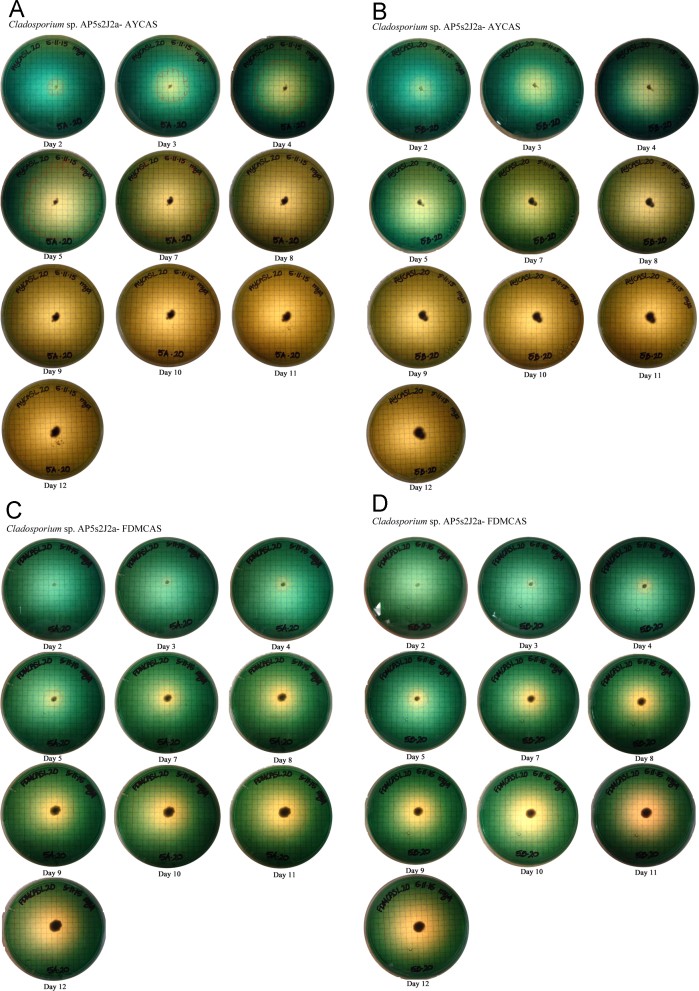
Image files of *Cladosporium* sp. AP5s2J2a grown on (A and B) Acetate–Yeast (AY) based CAS layer plates and (C &D) Fungal Defined Media (FDM) media based CAS layer plates. The text below each image indicates the time elapsed in days since inoculation. A. *Cladosporium* sp. AP5s2J2a – AYCAS. B. *Cladosporium* sp. AP5s2J2a – AYCAS. C. *Cladosporium* sp. AP5s2J2a – FDMCAS. D. *Cladosporium* sp. AP5s2J2a – FDMCAS.

**Fig. 5:**
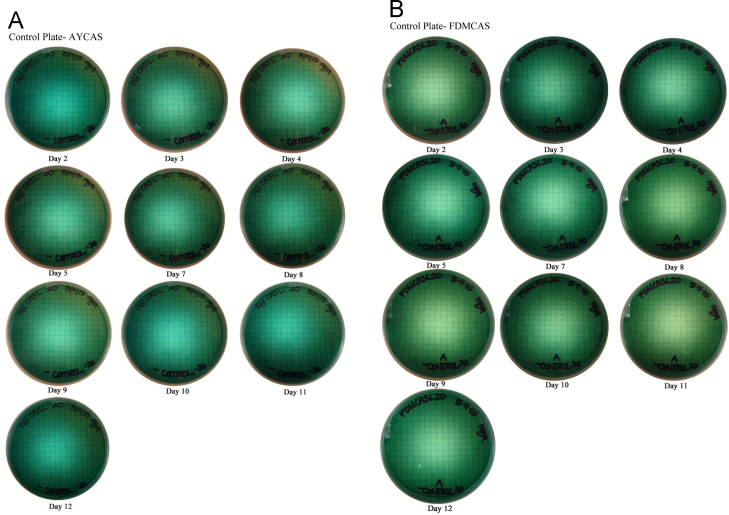
Images files of the negative control (A) AY CAS layer plates and (B) FDM CAS layer plates. These plates, which were made and imaged in the same manner as the inoculated fungal plates, verify that no color change occurred over time in uninoculated plates. A. Control Plate – AYCAS. B. Control Plate – FDMCAS.

This data in brief article provides the image data files supporting the method developed in [1], and provides clear examples and greater detail to aid in using the method. The image data included in Fig. 1 shows the systematic colorimetric change observed when a known amount of siderophore is added to the CAS assay layer plates. Fig. 2, Fig. 3, Fig. 4 show the visual color change observed due to the exudation of siderophores over time by fungi growing on CAS layer assay plates, and the lack of color change present in uninoculated plates (Fig. 5). These image sets are composed of photographs of colorimetric assay plates over several days, which have been analyzed using digital imaging software to calculate the area of the plates where the colorimetric change from blue to yellow has occurred. These areas are marked on each photo with a red line. These data are used in [1] to quantify fungal siderophore production and visualize exudation patterns.

## 2. Experimental design, materials and methods

The CAS assay, a colorimetric assay developed by Schwyn and Neilands [2] to measure the Fe-chelating function of siderophores (described in more detail in [1]), is a simple way to screen for siderophore production and measure quantities of siderophores produced. However, there are toxicity challenges in using this assay with fungal cultures due to the surfactants required for the assay to function. To address these challenges, we modified the original Schwyn and Neilands [2] colorimetric CAS assay by switching to a surfactant, N-Dodecyl-N,N-dimethyl-3-ammonio-1-propanesulfonate (DDAPS), shown to be less toxic [2] but with similar efficacy in the assay [3], and by creating “layer plates” which decreased the concentration of the toxic assay components at the growth surface of the agar plates.

The quantitative layer plates shown in the figures were made by first pipetting a 20 mL layer of growth media+CAS assay into 9 cm diameter petri dishes. Once completely solidified, a top layer of 20 mL of growth media only (no CAS assay) was added via pipette, and the plates were chilled overnight. The gradient resulting from the CAS assay diffusing upwards provides a lower toxicity growth surface for the fungi, while still functioning as a quantitative colorimetric assay in the plate as a whole.

We successfully used both Acetate–Yeast (AY) [1] and Fungal Defined Media supplemented with casamino acids (FDM) as growth media for these assay layer plates. The recipe for making the AYCAS layer plates is described in [1]. For the FDMCAS layer plates, the FDM top layer was made by autoclaving 0.0098 g MgSO_4_, 4.1015 g sodium acetate, and 6.607 g ammonium sulfate in 880 mL deionized water. Once cooled to 60 °C, 100 mL of 6.45 mM K_2_HPO_4_ solution, 20 mL of 1 M HEPES solution, and 1 mL trace elements solution, all filter-sterilized, were added. This media was supplemented with 1 ml of a filter-sterilized 10 g/100 ml casamino acids solution to provide potential precursor amino acids for siderophore production. The FDMCAS bottom layer was created as described above, but using only 770 mL of deionized water for the autoclaved portion of the media, and adding 100 mL 10X CAS post-autoclaving. Layers were pipetted using a 10 mL pipette with sterile tip, in a sterile flow hood.

We also tried a third media (5 g glucose, 0.01 g yeast extract, 0.5 g KH_2_PO_4_, 0.15 g MgSO_4_, 2.5 mL of 10% (NH_4_)_2_HPO_4_ solution, 5 mL of 1% CaCl_2_ solution, and 2.5 mL of 1% NaCl solution, made up to 1 L total with deionized water) which gave inconsistent results, and often produced orange, purple, and gray color changes, the meaning of which is unclear (data not shown).

In Fig. 1, we can see the color change associated with adding known quantities of a siderophore, desferrioxamine B (DFOB), as described in [1]. These data were used to both verify and calibrate the quantitative assay plates. It is important to allow the DFOB solution to fully diffuse over several hours, preferably overnight, to get correct results. Only allowing the added DFOB to diffuse and react for 1-2 hours produced an underestimation of the amount of siderophore present.

In Fig. 2, Fig. 3, Fig. 4, three fungal isolates (described in Santelli et al. [4], [5]) were inoculated onto plates as described in [1]. The inoculated plates, along with the uninoculated negative control plates (Fig. 5), were sealed with parafilm, and incubated at 25 °C in the dark for 12 days.

**Fig. 6:**
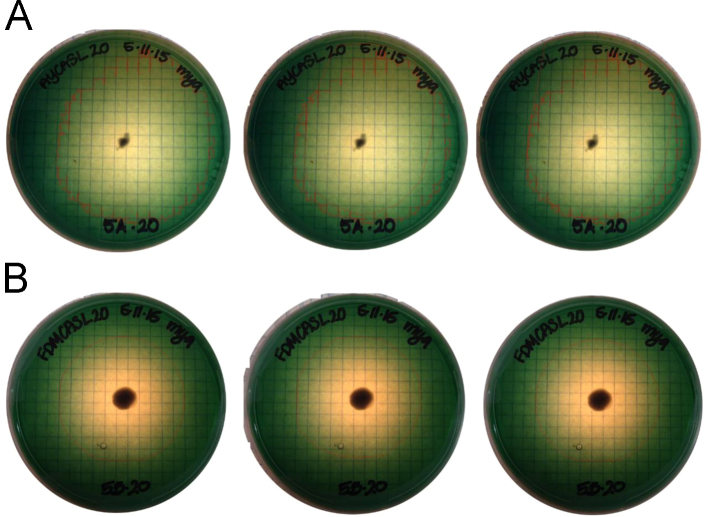
To assess the consistency of the digital data analysis protocol, we conducted replicate analyses on the same plates and compared the results. Examples of triplicate analyses on the same plate of each media type are shown for (A) *Cladosporium* sp. AP5s2J2a AY grown on imaged on Day 5 and B) *Cladosporium* sp. AP5s2J2a grown on FDMCAS on imaged Day 11. For each triplicate analysis, the resulting calculated siderophore equivalencies varied less than 10% between the replicates.

To collect the data presented here, photos of each plate were taken on a lightbox, daily for 12 days. Images were cropped to remove background and resized in Adobe Photoshop. The image files were scaled to 9 cm using the spatial calibration tool in JMicroVision 1.2.7. [6], ensuring the digitally measured area would match the actual plate area, and then the magic wand tool, under the object extraction menu, was used to select the yellow color of the deferrated CAS assay on the plates. We picked the yellow at the outer edge of the visible area of color change, checked the “fill hole” option, and then adjusted the luminosity, hue, and saturation sliders until the entire yellow area was selected. We have found that in general, selecting the “convex hull” option gives good results, but this should be used in conjunction with the other options already described to satisfactorily delineate the area of color change on the assay plate photographs. Once the entire area was selected, we could “add selection” from the bottom of the magic wand menu screen and retrieve the measured area value from the data viewer icon located above the image. By multiplying the measured area by the amount of CAS assay present per unit area, this method provides a quantitative estimate of the siderophore equivalents produced [1]. To check the reproducibility of the area measurement technique, four plates were analyzed three times each resulting in less than 10% variation (Fig. 6).

**Fig. 7:**
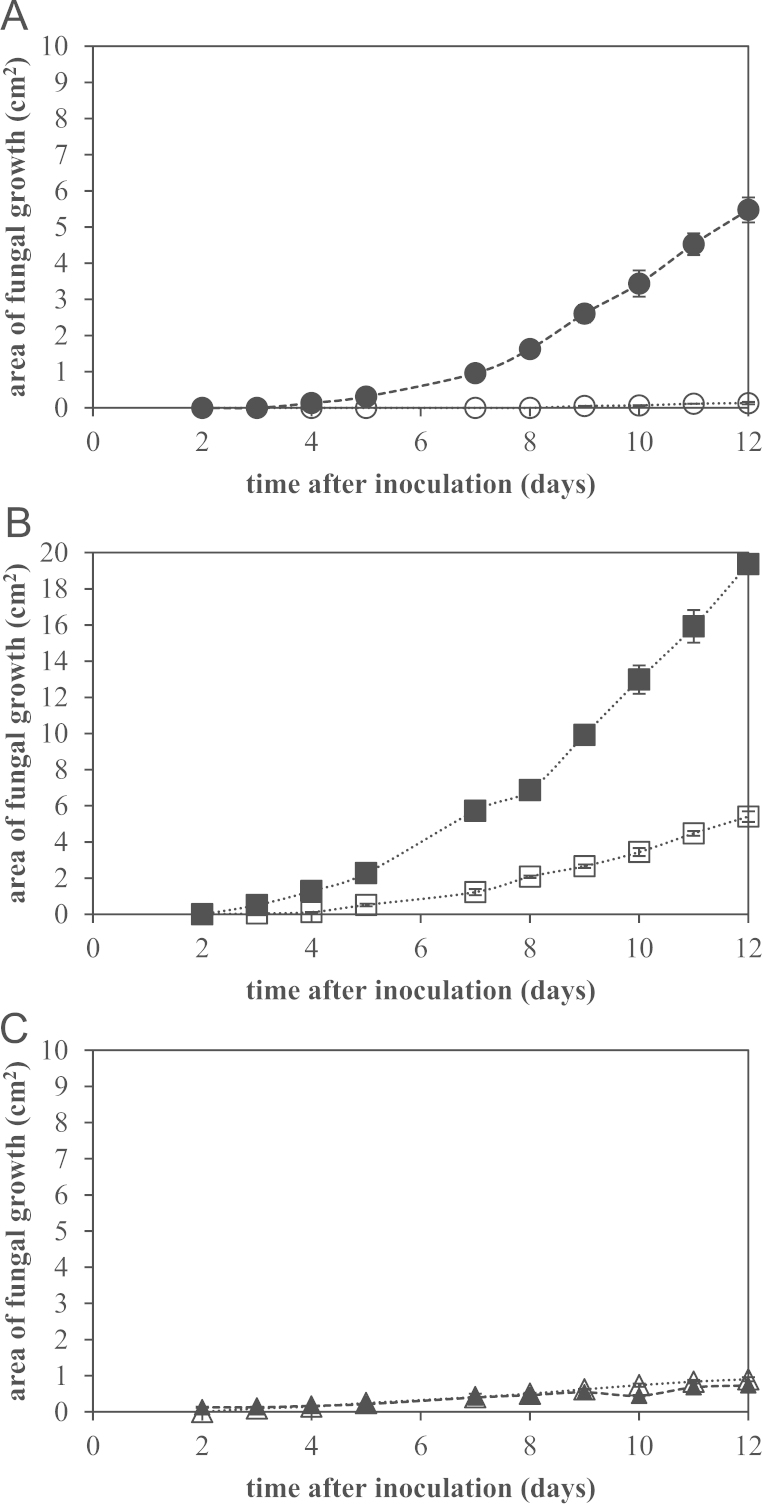
Comparison of growth area (cm^2^) of the three fungal isolates on duplicate AYCAS (filled symbols) and FDMCAS (open symbols) layer plates plates over time; A: circles=*Pyrenochaeta* sp. AP1s5JAC1a, B: squares=*Paraconiothyrium* sp. AP3s5J2a, and C: triangles=*Cladosporium* sp. AP5s2J2a. Error bars are standard error; where error bars are not visible, they are smaller than the data marker. Lines are meant to guide the eye only.

In addition to quantifying siderophore exudation, we also used similar methods to digitally quantify fungal growth area on the layer plates (Fig. 7). For pigmented or non-spherical fungal colonies, we used the magic wand tool method described above. For pale fungi, which are difficult to differentiate from the plate colors, or circular colonies, we used the 2-D measurement tool rather than the magic wand tool.
