# Supplementary material for: Digital image quantification of siderophores on agar plates
Source: Data Brief. 2016 Feb 3;6:890–8. doi: 10.1016/j.dib.2016.01.054 (PMC4752732; doi:10.1016/j.dib.2016.01.054)
Supplement: Supplementary file 1 — Supplementary material [file mmc1.doc]

We wish to confirm that there are no known conflicts of interest associated with this publication and there has been no significant financial support for this work that could have influenced its outcome.


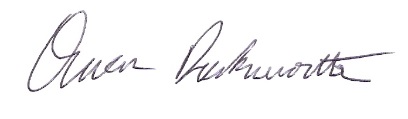


Owen Duckworth

Jaunary 19^th^, 2016
